# Bilateral lung transplantation for pulmonary emphysema associated with cutis laxa

**DOI:** 10.1016/j.jhlto.2024.100086

**Published:** 2024-03-20

**Authors:** Akira Matsumoto, Akihiro Ohsumi, Tomohiro Handa, Susumu Sato, Hiroyuki Katsuragawa, Hiroshi Date

**Affiliations:** a Department of Thoracic Surgery, Kyoto University Hospital, Kyoto, Japan; b Department of Advanced Medicine for Respiratory Failure, Kyoto University Hospital, Kyoto, Japan; c Department of Respiratory Care and Sleep Control Medicine, Kyoto University Hospital, Kyoto, Japan; d Department of Diagnostic Pathology, Kyoto University Hospital, Kyoto, Japan

**Keywords:** cutis laxa, lung transplantation, elastic fiber disorder, pulmonary emphysema, lobar bronchial anastomosis

## Abstract

Cutis laxa is a rare elastic tissue disorder that mainly affects the skin and results in loss of elasticity. Occasionally, pulmonary emphysema complicates this condition. Herein, we report the first case of successful lung transplantation for severe juvenile emphysema associated with cutis laxa. The patient underwent bilateral deceased-donor lung transplantation. The patient’s lungs had very thin visceral pleura due to a deficiency of elastic fibers along with bronchomalacia and fragile bronchial cartilage. The right upper bronchus and truncus intermedius were individually anastomosed to improve bronchial healing. At the 6-month follow-up, bronchoscopy revealed successful healing of all bronchial anastomoses, and the patient’s quality of life improved. Lung transplantation is effective for treating pulmonary emphysema caused by cutis laxa.

## Background

Cutis laxa is a rare connective tissue disease caused by a disordered elastic fiber network leading to loss of skin elasticity. Although the life expectancy of patients with cutis laxa is generally normal, other internal organs may be affected. Severe emphysema can result in poor pulmonary function. This is the first report of a patient with cutis laxa complicated by emphysema who required lung transplantation.

### Case presentation

**Figure 1:**
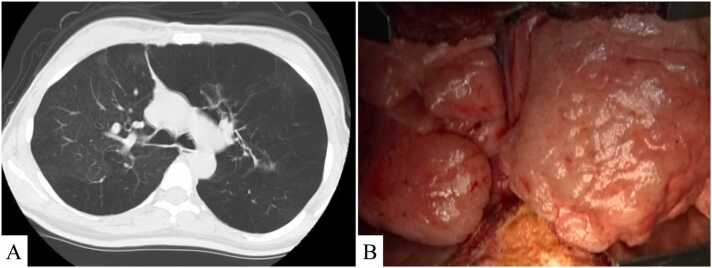
Computed tomography revealed emphysema and lung hyperinflation before transplantation (A). Intraoperatively, the recipient's lungs lacked elasticity (B).

**Figure 2:**
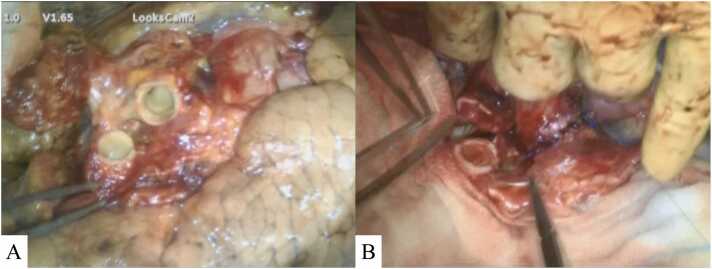
The upper lobe bronchus and bronchus intermedius of the right donor lung were divided and trimmed short (A). The bronchus intermedius was anastomosed first, and the recipient's upper lobe bronchus was trimmed to facilitate anastomosis (B).

**Figure 3:**
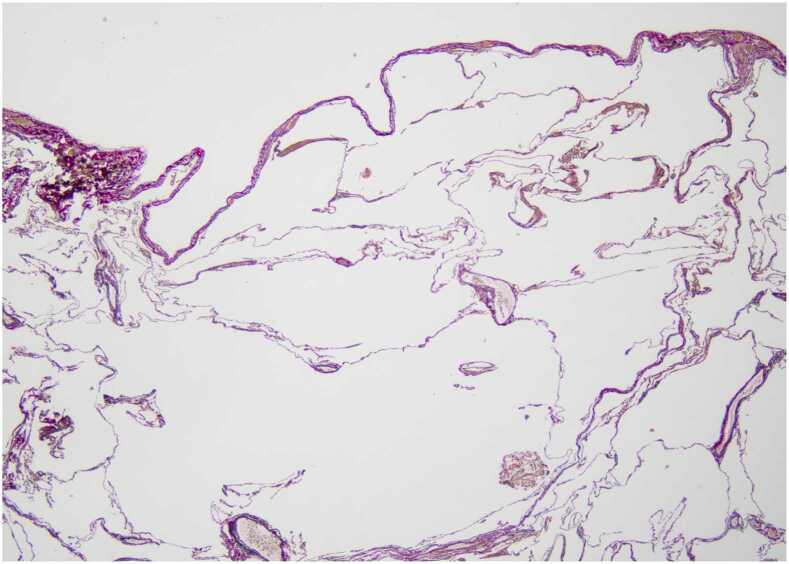
Elastica van Gieson stained microscopic image of the recipient’s lung near the visceral pleura. The alveolar space was diffusely enlarged and the alveolar wall was thin. Elastic fibers had a less prominent reticular structure than normal and disappeared in the visceral pleura.

A previously healthy 11-year-old girl presented with reduced skin elasticity and progressive dyspnea. At the age of 19 years, she was diagnosed with cutis laxa and juvenile pulmonary emphysema. She had a severe allergic reaction to antibiotics before the onset of cutaneous symptoms, which appeared to be related to the subsequent development of cutis laxa. No other organs were affected. Cutaneous symptoms were treated with multiple plastic surgeries. The patient was referred to our hospital at 26 years of age as a potential recipient of deceased-donor lung transplantation. At registration, the patient’s forced expiratory volume in 1 second was 0.55 liter (18.5% of the predicted volume), which dropped to 0.37 liter (12.7% of the predicted volume) after 2 years. After waiting 3 years, the patient underwent bilateral deceased-donor lung transplantation with venoarterial extracorporeal membrane oxygenation support (Figure 1). The recipient’s bronchus was found to exhibit bronchomalacia with very thin and fragile bronchial cartilage during the surgery. The right upper bronchus and truncus intermedius of the donor right lung graft were individually divided at the back table and anastomosed with the recipient’s bronchi to improve the retrograde bronchial blood supply to the right graft (Figure 2). Left bronchial anastomosis was conventionally performed at the level of the left main bronchus. A pathological examination revealed an absence of elastic fibers in the visceral pleura (Figure 3). The bronchi and vessels showed no pathological abnormalities. At the 6-month follow-up, bronchoscopy showed successful healing of all bronchial anastomoses; forced expiratory volume in 1 second was improved to 2.43 liter (84.1% of the predicted volume) and the patient’s quality of life improved.

## Discussion

Cutis laxa is a disorder of the elastic fiber network. Cutaneous symptoms and loss of elasticity can be treated with plastic surgery. However, other internal organs, such as the lungs, may also be affected, leading to emphysema and poor prognosis.^1^, ^2^ Currently, there is no standardized treatment for the pulmonary complications of cutis laxa.

Here, we reported the case of lung transplantation for pulmonary emphysema associated with cutis laxa. Although cutis laxa does not affect wound healing after plastic surgery, it was unclear whether it also applies to the healing of bronchial anastomoses in a post-transplant immunosuppressive state. Furthermore, similar to the previous report of tracheomalacia with cutis laxa,^3^ the recipient’s bronchus was fragile and had a higher risk of developing airway complications. In general, airway complications are more common in the right lung, and technically, shortening the donor bronchus to preserve the retrograde bronchial blood supply has been effective in preventing complications.^4^, ^5^, ^6^ In this patient, to improve the bronchial blood supply for better bronchial healing, the right donor bronchi were divided into the right upper bronchus and truncus intermedius to minimize the donor bronchus and individually anastomosed. Considering the ischemic time and low complication rate, left bronchial anastomosis was conventionally performed at the level of the left main bronchus. Post-transplantation, all 3 bronchial healings were satisfactory and the patient’s quality of life markedly improved.

In conclusion, we report the successful case of lung transplantation in a patient with cutis laxa complicated with juvenile emphysema. Although the patient had elastic fiber deficiency in the visceral pleura and the patient’s bronchial cartilage was very thin and fragile, individual bronchial anastomosis of the right upper bronchus and truncus intermedius resulted in satisfactory bronchial healing.

## Consent for publication

The authors declare that appropriate written informed consent was obtained for the publication of this manuscript and the accompanying images.

## Disclosure statement

The authors declare that they have no known competing financial interests or personal relationships that could have appeared to influence the work reported in this paper.

The authors have no funding sources to disclose.

## Data Availability

Data supporting the study findings are available from the corresponding author upon reasonable request.

## References

[1] Berk D.R., Bentley D.D., Bayliss S.J. (2012). Cutis laxa: a review. J Am Acad Dermatol.

[2] Turner Stokes L., Turton C., Pope F.M., Green M. (1983). Emphysema and cutis laxa. Thorax.

[3] Urban Z., Hucthagowder V., Schürmann N. (2009). Mutations in LTBP4 cause a syndrome of impaired pulmonary, gastrointestinal, genitourinary, musculoskeletal, and dermal development. Am J Hum Genet.

[4] Yserbyt J., Dooms C., Vos R. (2016). Anastomotic airway complications after lung transplantation: risk factors, treatment modalities and outcome-a single-centre experience. Eur J Cardiothorac Surg.

[5] van Berkel V., Guthrie T.J., Puri V. (2011). Impact of anastomotic techniques on airway complications after lung transplant. Ann Thorac Surg.

[6] Crespo M.M., McCarthy D.P., Hopkins P.M. (2018). ISHLT consensus statement on adult and pediatric airway complications after lung transplantation: definitions, grading system, and therapeutics. J Heart Lung Transplant.

